# Label-free quantification of host cell protein impurity in recombinant hemoglobin materials

**DOI:** 10.1007/s00216-023-05024-8

**Published:** 2023-11-26

**Authors:** André Henrion, Cristian-Gabriel Arsene, Maik Liebl, Gavin O’Connor

**Affiliations:** 1 Physikalisch-Technische Bundesanstalt (PTB), Bundesallee 100, 38116 Braunschweig, Germany; 2 Department of Biochemistry and Bioinformatics, Technische Universität Braunschweig, 38106 Braunschweig, Germany

**Keywords:** Label-free quantification, Protein quantification, Hemoglobin-A2 (HbA_2_) protein, Protein reference materials

## Abstract

Quantitative analysis relies on pure-substance primary calibrators with known mass fractions of impurity. Here, label-free quantification (LFQ) is being evaluated as a readily available, reliable method for determining the mass fraction of host cell proteins (HCPs) in bioengineered proteins which are intended for use as protein calibration standards. In this study a purified hemoglobin-A2 (HbA_2_) protein, obtained through its overexpression in *E. coli,* was used. Two different materials were produced: natural and U^15^N-labeled HbA_2_. For the quantification of impurities, precursor ion (MS1-) intensities were integrated over all *E. coli* proteins identified and divided by the intensities obtained for HbA_2_. This ratio was calibrated against the corresponding results for an *E. coli* cell lysate, which had been spiked at known mass ratios to pure HbA_2_. To demonstrate the universal applicability of LFQ, further proteomes (yeast and human K562) were then alternatively used for calibration and found to produce comparable results. Valid results were also obtained when the complexity of the calibrator was reduced to a mix of just nine proteins, and a minimum of five proteins was estimated to be sufficient to keep the sampling error below 15%. For the studied materials, HbA_2_ mass fractions (or purities) of 923 and 928 mg(HbA_2_)/g(total protein) were found with expanded uncertainties (*U*) of 2.8 and 1.3%, resp. Value assignment by LFQ thus contributes up to about 3% of the overall uncertainty of HbA_2_ quantification when these materials are used as calibrators. Further purification of the natural HbA_2_ yielded a mass fraction of 999.1 mg/g, with a negligible uncertainty (*U* = 0.02%), though at a significant loss of material. If an overall uncertainty of 5% is acceptable for protein quantification, working with the original materials would therefore definitely be viable, circumventing the need of further purification.

Protein quantification by mass spectrometry (MS) is considered to make an essential contribution to strategies toward precision diagnostics [1]. Basically, uncertainties of 5%, or less, can be achieved with proteins if isotope labeled internal standards are employed (ID-MS) [2]. However, a lack of information about the impurity fraction in the calibrator material increases the overall uncertainty and may contribute to the lack of reproducibility and/or comparability of measurement results. Methods and approaches have just recently been reviewed for impurity determination in organic compounds intended for use as primary calibrators in quantitative analysis [3].

Rather than looking at small organic molecules, the present work is motivated by the additional need for well-characterized reference materials (RMs) for the targeted quantification of proteins. Depending on the measurement strategy involved, either proteotypic peptides or full length proteins are used for calibration [4–6]. For peptides, different approaches to impurity measurement have been studied, as was reviewed in Josephs et al. [7]. Direct quantification by amino acid analysis (AAA), quantitative nuclear resonance spectroscopy (qNMR), or elemental analysis was found to work best in many situations. To obtain accurate results, rigorous detection, quantification, and correction for interfering compounds are required [8–10]. A complementary approach consists of the one-by-one detection, identification, and quantification of individual contaminants as separate analytes to obtain the mass fraction of the impurity. Such mass-balance approaches, in spite of being labor-intensive, are a viable and common option for short peptides. Typically, solid-phase synthesis (SPSS) is used for their production. The main routes and causes of deviation from the intended amino acid sequence are well known for SPSS [11, 12]. In such a setting, therefore, the number of contaminants to be taken into account may be small and manageable.

In contrast to this, the practicality of both mentioned approaches is complicated when used for determining the purity of protein materials, if possible at all. Indeed, effective methods are available for removing host cell-related proteins (HCPs) from the target after expression. Still, there are circumstances that may cause significant amounts of HCPs to remain in the product. For example, in *E. coli*, this has been pointed out to typically happen if the expression yield is low [13]. Overexpression of the target might also induce expression of a number of bacterial proteins due to pleiotropism and/or stress conditions. There are recurring basic patterns of such proteins, as identified in Bolanos-Garcia and Davies [13]. These are confined to a much smaller subset compared to the original proteome. Although this reduces their number, the presence and individual abundances of HCPs may vary between preparations. In many instances, residing HCPs will still be many in number, thus limiting the practicability of the one-by-one approach.

Here, we systematically evaluate the use of label-free quantification (LFQ) of proteins by precursor-ion (MS1) intensities to reliably obtain the mass fraction of HCPs in a given sample. Central to the functioning of LFQ is that an amount of any peptide produces a specific amount of MS1 intensity per mass of protein regardless of what the individual peptides (proteins) are. Unlike in most applications of LFQ, [5, 6, 14, 15] in our context, peptide intensities are not collected separately per protein, but are rather integrated over all proteins identified from the host cell proteome. We demonstrate that this quantity can be calibrated against known amounts of the host cell proteome. Beyond this, it will be shown that any other proteome, or even a protein mixture of just a few common proteins, could likewise be used for calibration. The example presented here is hemoglobin-A2 (HbA_2_), a pure-substance material obtained by overexpression in *E. coli*. The material was produced as a primary calibrator for the quantification of the mass fraction of HbA_2_ in whole blood by isotope dilution mass spectrometry (ID-MS) [16, 17]. Besides the natural form, a U-^15^N-labeled version was also engineered, thus providing an internal standard. In either case, purification by immobilized metal affinity chromatography (IMAC) had left an estimated 5–10% mass fraction of *E. coli* proteins. In addition to the (natural) HbA_2_ material and the (labeled) U-^15^N-material, a third material was included in the study. This was obtained through further purification of the natural HbA_2_ material (referred to as ultra-purified HbA_2_). This was then used to demonstrate the applicability of the approach to low-level impurity materials as well.

## Experimental section

### Study materials

Recombinant HbA_2_ (*α*_2_*δ*_2_) in natural and U-^15^N-labeled form was kindly provided by the European Commission, Joint Research Centre, Geel, Belgium. The materials were produced by Trenzyme GmbH as previously described [17] using P69905 and P02042 (UniProtKB) as templates for the co-expression in *E. coli* of the *α* and *δ* subunits, respectively. Both materials were obtained as solutions of about 0.42 mg/g (natural form) and 0.47 mg/g (labeled form) in 50 mmol/L 2-Amino-2-(hydroxymethyl)propane-1,3-diol (Tris), pH 7.5, and 100 mmol/L NaCl. The sample volumes were 28 mL (HbA_2_) and 26 mL (U-^15^N-HbA_2_). The recombinant HbA_2_ (*α*_2_*δ*_2_) of natural isotopic composition was ultra-purified by semi-preparative strong anion exchange chromatography using a MONO-Q 4.6/100 PE column, yielding 8 mL of ultrapurified HbA_2_ at a concentration of 0.43 mg/g. This was then used as the third study material. All study materials were stored in a fridge at 4–8 °C until used.

#### Calibrators

##### HbA_2_ used for preparation of the calibrators

**Fig. 1:**
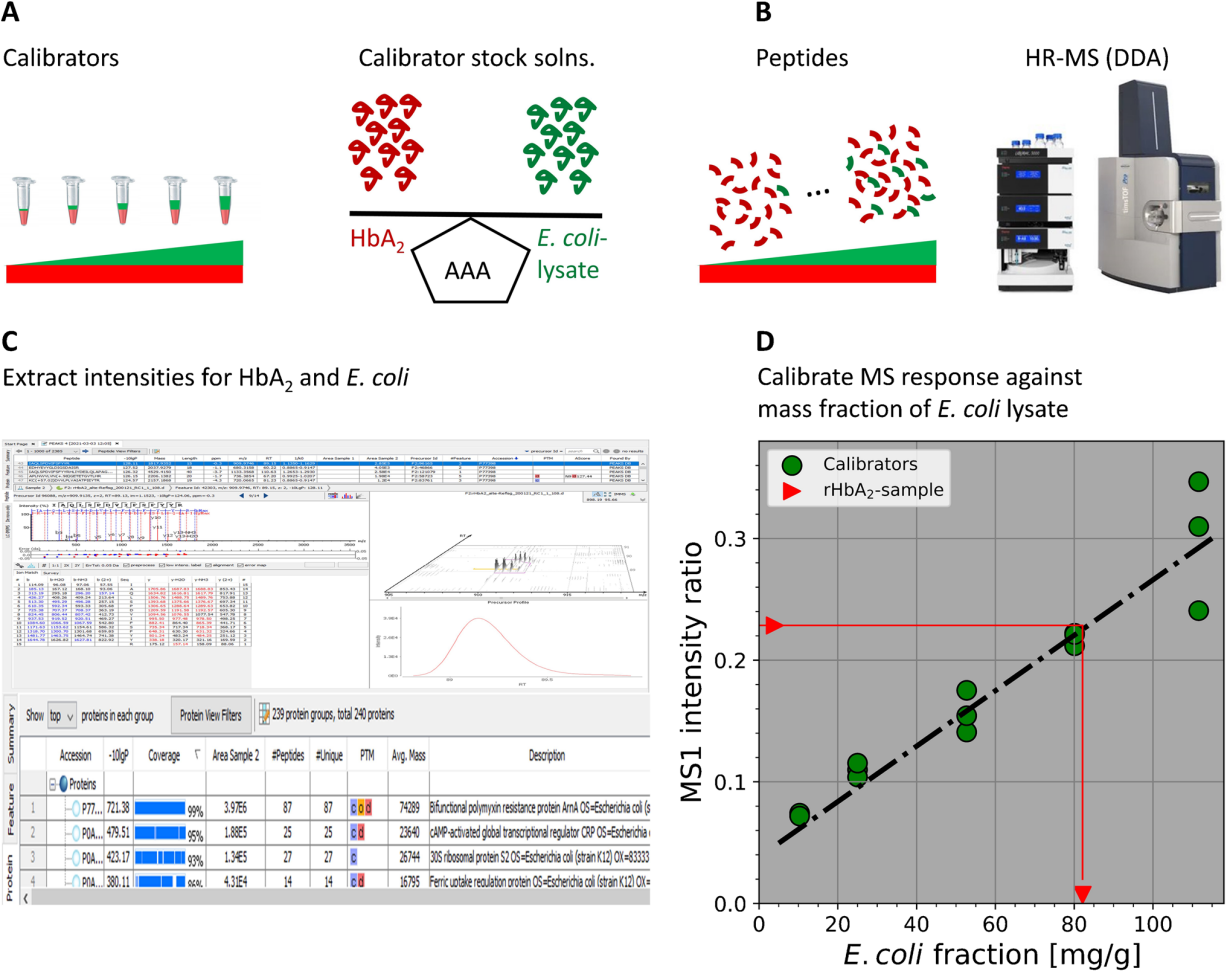
LFQ-based measurement of HCPs (*E. coli*) mass fraction in recombinant HbA_2_. **A** Calibrators were obtained by spiking aliquots of HbA_2_ stock solution (red) with increasing amounts of *E. coli* lysate (green). Amounts per mass (mg) of both components (HbA_2_ and *E. coli*) and mass fractions of *E. coli* were hence known for each calibrator through amino acid analysis. **B** Quantitative information was acquired by shotgun proteomics. **C** MS1 intensities were integrated over all features associated with peptides identified from either *E. coli* or HbA_2_. **D** The sum of all MS1 peak intensities from peptides associated with *E. coli* per sum of all peak intensities associated with HbA2 peptides for the calibrators were plotted vs. mass fractions. The fitting linear function was then used to calculate the *E. coli* fraction in the investigated materials from sample measurements (red line and arrows)

The material was obtained from SIGMA-Aldrich, cat. No.: H0266; lot: SLBK8749V, as a neat substance. The “protein-purity” was 99.0% using the LFQ method described herein. For value-assignment, a stock solution was prepared from this solid material by dissolving ∼2 mg in 1 g of Tris (10 mmol/L, pH 7.8). The mass fraction of HbA_2_ in the solid material was 482.7 mg/g as determined by AAA. A stock solution of the HbA_2_ material in Tris (10 mmol/L, pH 7.8) was prepared. An aliquot of this was used as a constant component present in each of the calibrators (red in Fig. 1A).

##### *E. coli* proteome sample

Lyophilized *E. coli* protein material was obtained from BIO-RAD (ReadyPrep, Catalog 163 2110, L9703999, Control 310,004,134). For a stock solution, the material was reconstituted in water (30% acetonitrile, 0.1% formic acid); the mass fraction of *E.coli* proteins as determined by AAA in that stock solution was 0.345 ± 0.012 mg/g. A series of calibrator samples was prepared by mixing an aliquot of HbA_2_ stock solution with the appropriate amount of *E. coli* stock solution (red and green, respectively, in Fig. 1A). The mass fractions of HbA_2_ in these sample solutions were 0.390, 0.383, 0.372, 0.361, and 0.350 mg/g (solution). The corresponding mass fractions of *E. coli* protein, relative to HbA_2_ in the calibrator samples, was 10.3, 25.0, 52.7, 80.1, and 111.6 mg (*E. coli*)/g(HbA2).

##### Yeast proteome sample

Yeast-based calibrators were prepared in the same way as described for *E. coli*. A whole-cell protein extract of *Saccharomyces cerevisi*ae (Promega, V7341, lot 434,786) was deployed. The material came as a solution in 50 mmol/L Tris and 6.5 mol/L urea. Solutions containing mass fractions of HbA_2_ (0.390, 0.387, 0.383, 0.378, and 0.372 mg/g) were prepared. Mass fractions of yeast proteins in these solutions were 12.7, 26.5, 52.4, 76.6, and 113.6 mg/g (relative to HbA_2_).

##### Human K562 proteome sample

A whole-cell protein extract from human K562 cells (Promega, V6941, lot 444,583) was dissolved in 50 mmol/L Tris and 6.5 mol/L urea, as above. Solutions containing mass fractions of HbA_2_ (0.390, 0.387, 0.383, 0.378, and 0.372 mg/g) were prepared, comprising also K562-proteins at 15.0, 32.7, 63.9, 94.3, and 136.2 mg/g as mass fractions relative to HbA_2_.

##### Protein mix

Human C-reactive protein (CRM GBW09228, National Institute of Metrology, China), human insulin analog (insulin aspart, NovoLog) and human *β*2-microglobulin (kindly provided by the European Commission, Joint Research Centre, Geel, Belgium) were obtained as solutions. Bovine serum albumin (Sigma-Aldrich, cat. No. 05470, lot No. 1099572), myoglobin from horse skeletal muscle (Sigma-Aldrich, cat. No. 70025, lot No. 381848/1), cytochrome-c from bovine heart (Sigma-Aldrich, cat. No. C3131, lot SLBZ0555), somatotropin (NIBSC, WHO International Standard 98/574), human ceruloplasmin (Athens Research & Technology, cat. No. 16–16-030518), and serotransferrin (Sigma-Aldrich, cat. No. T3309, lot BCBR1763V) were obtained as solids and had to be dissolved to known concentrations in water prior to use. The mass fractions of somatotropin, ceruloplasmin, serotransferrin, *β*2-microglobulin, and insulin were determined by mass spectrometry based AAA, while certified values were used, as provided by the supplier, for C-reactive protein, albumin, cytochrome-c, and myoglobin. Aliquots of these solutions were mixed to yield a stock solution containing somatotropin, ceruloplasmin, serotransferrin, *β*2-microglobulin, insulin, C-reactive protein, albumin, cytochrome-c, and myoglobin in the mass-ratio of 0.1055:0.1176:0.124:0.1251:0.1247:0.0317:0.1215:0.1260:0.1233. Aliquots of this mixed solution were spiked with aliquots of the HbA_2_ stock solution, resulting in HbA_2_ mass fractions of 0.393, 0.388, 0.383, 0.378, 0.372, and 0.367 mg/g, and protein mass fractions, relative to HbA_2_, of 22.7, 47.4, 69.9, 92.8, 117.1, and 140.8 mg/g. To additionally cover the low HCPs fraction range as needed for the ultra-purified HbA_2_ material, a second series of calibrator samples was prepared. These calibrators were of 0.427 mg/g HbA_2_ mass fraction and 0.2, 0.3, 0.4, 0.7, 1.1, and 1.4 mg/g protein mass fractions relative to HbA_2_.

### Determination of protein mass fractions in the calibrators

For the stock solutions used to prepare the calibrators, the mass fractions of amino acids were determined by mass spectrometry based AAA, as detailed in Arsene et al. [17]. These mass fractions were then combined with the known mass fractions (or relative amounts) of these amino acids in the protein or proteome to yield the protein mass fraction in that stock solution. In the cases of *E. coli*, yeast and K562, relative amounts (by mass) of amino acids were used, as was previously published [18–20]. The contribution to the overall estimated measurement uncertainty from AAA (in our laboratory) and uncertainties published with literature data were combined to yield expanded (95%) uncertainties of 3.5%, 2.7%, 3.0%, and 3.2%, respectively, for the *E. coli*, yeast, K562, and protein-mix calibrator stock solutions.

### Proteolysis

To a 30 *µ*L aliquot of sample (recombinant HbA_2_) or calibrator, 70 *µ*L of Tris solution (35 mg Tris base, 46 mg Tris HCl, dissolved in 1 mL water) were added. Proteolysis (37 °C) was started by the addition of 10 *µ*L of trypsin solution (1 mg/mL in 50 mM acetic acid). Trypsin from porcine pancreas was obtained from Sigma-Aldrich, St Louis, USA; cat. No.: T0303. After 10, 70, 130, 190, and 250 min, further 10 *µ*L aliquots of trypsin solution were added. In parallel, 40 *µ*L aliquots of acetonitrile were added after 10, 30, 60, 90, 120, and 150 min, respectively. The sample or calibrator was further incubated at 37 °C overnight. For reduction, 0.8 mg of dithiothreitol (DTT) was added. After incubation (37 °C) for 1 h, 3 mg of 2-iodoacetamide were added for alkylation (30 min at room temperature). The excess of 2-iodoacetamide was quenched with 3 mg of DTT. The reaction was stopped by the addition of 10 *µ*L of formic acid (10 vol.-%). The sample or calibrator was desalted using solid-phase extraction (SPE) C18 ec cartridges (Chromabond, 100 mg, Macherey Nagel, Düren, Germany). After lyophilization, residues were redissolved in 40 *µ*L of water (0.1% formic acid) and subjected to nLC-MS/MS analysis.

### Liquid chromatography-mass spectrometry

An UltiMate 3000 RSLCnano HPLC system (Thermo Fisher Scientific) coupled to a timsTOF Pro mass spectrometer (Bruker Daltonics) was used for the analysis of the proteolysed samples and calibrators. Peptides were trapped on a pre-column (Acclaim PepMap C18, 5 µm, 0.3 × 5 mm) and then separated on a Bruker Fifteen nanoFlow column (15 cm × 75 µm, C18, 1.9 µm, 120 Å) using a linear water-acetonitrile gradient from 1 to 60% B in 210 min and then from 60 to 80% B in 20 min (with solvent A: water, 0.1 vol.-% formic acid and B: acetonitrile, 0.1 vol.-% formic acid) at 40 °C. The flow rate was 300 nl/min. The timsTOF Pro mass spectrometer was equipped with a CaptiveSpray ion source. The mass spectrometer was run using the DDA-PASEF-standard-1.1 s-cycle time method, as provided by Bruker. Briefly, the settings were 10 PASEF MS/MS scans per acquisition cycle with a trapped ion mobility accumulation and elution time of 100 ms. Spectra were acquired in a *m/z* range of 100 to 1700 and in an (inverse) ion mobility range (1*/K*_0_) of 0.60 to 1.60 Vs/cm^2^. The collision energy was set up as a linear function of ion mobility starting from 20 eV for 1*/K*_0_ of 0.6 to 59 eV for 1*/K*_0_ of 1.6.

### Protein database search

PEAKS Studio Xpro (Bioinformatics Solutions Inc.) was used for feature detection/database searching and precursor ion (MS1) quantification. Databases for *E. coli*, *Saccharomyces cerevisiae*, human proteome, and the mixture of nine proteins were obtained as FASTA files (uniprot.org, accessed: 27. Aug. 2021). FASTA files of human hemoglobin subunit alpha and delta (Uniprot: P69905 and P02042) were added to the databases of non-human proteomes. The following settings were applied for data analysis: carbamidomethylation of cysteine as fixed modification, methionine oxidation, and glutamine or asparagine deamidation as variable modifications. A maximum of two modifications per peptide were allowed. With the human proteome, glycosylation was set as an additional variable modification using the built-in glycosylation list. Trypsin/P was set as the enzyme, and no more than two missed cleavages per peptide were allowed. The mass tolerance for the monoisotopic mass of precursor ions and fragment ions was 15 ppm and 0.05 Da, respectively. For the retention time and ion-mobility of an identified peptide, the shift tolerance between different runs was 3 min and 5%, respectively. Mass correction was enabled for precursor ions. The false discovery rate (FDR) was 1% at the peptide and protein level. The minimum length of identified peptides was seven amino acids. Results of quantification were obtained as peak areas at the protein-level.

### Mass fraction of impurity in the labeled HbA_2_ material

The mass fraction of impurity in the labeled HbA_2_ material was 78.1 ± 8.6 mg/g. The individual results from *n* = 6 repetitions of label-free quantification were 70.8, 72.4, 84.2, 92.8, 73.2, and 75.0 mg/g.

### Fractions of co-purifying proteins

The fraction of *E. coli* proteins known to frequently be co-purified [13] was calculated as the ratio of the (MS1) intensity of these proteins to the intensity of all *E. coli* proteins identified in the HbA_2_ material or in the *E. coli* proteome sample. For Fig. 3, fractions were calculated for the two HbA_2_ materials and compared to the fraction for an *E. coli* proteome sample containing a similar amount of *E. coli* proteins (80.1 mg/g).

### Downstream data analysis

Further analysis of the data exported from PEAKS was based on Python 3.8 with the modules pandas, numpy, numpy.linalg, and Matplotlib imported as needed. For datafit and cross-validation, Scikit-learn [21] 1.0.2 was used.

### Data availability

The mass spectrometry data and the tables of identified and quantified proteins have been deposited to the ProteomeXchange Consortium via the PRIDE partner repository with the dataset identifier PXD041736.

## Results and discussion

### Calibration and value assignment to the HbA_2_ materials

**Fig. 2:**
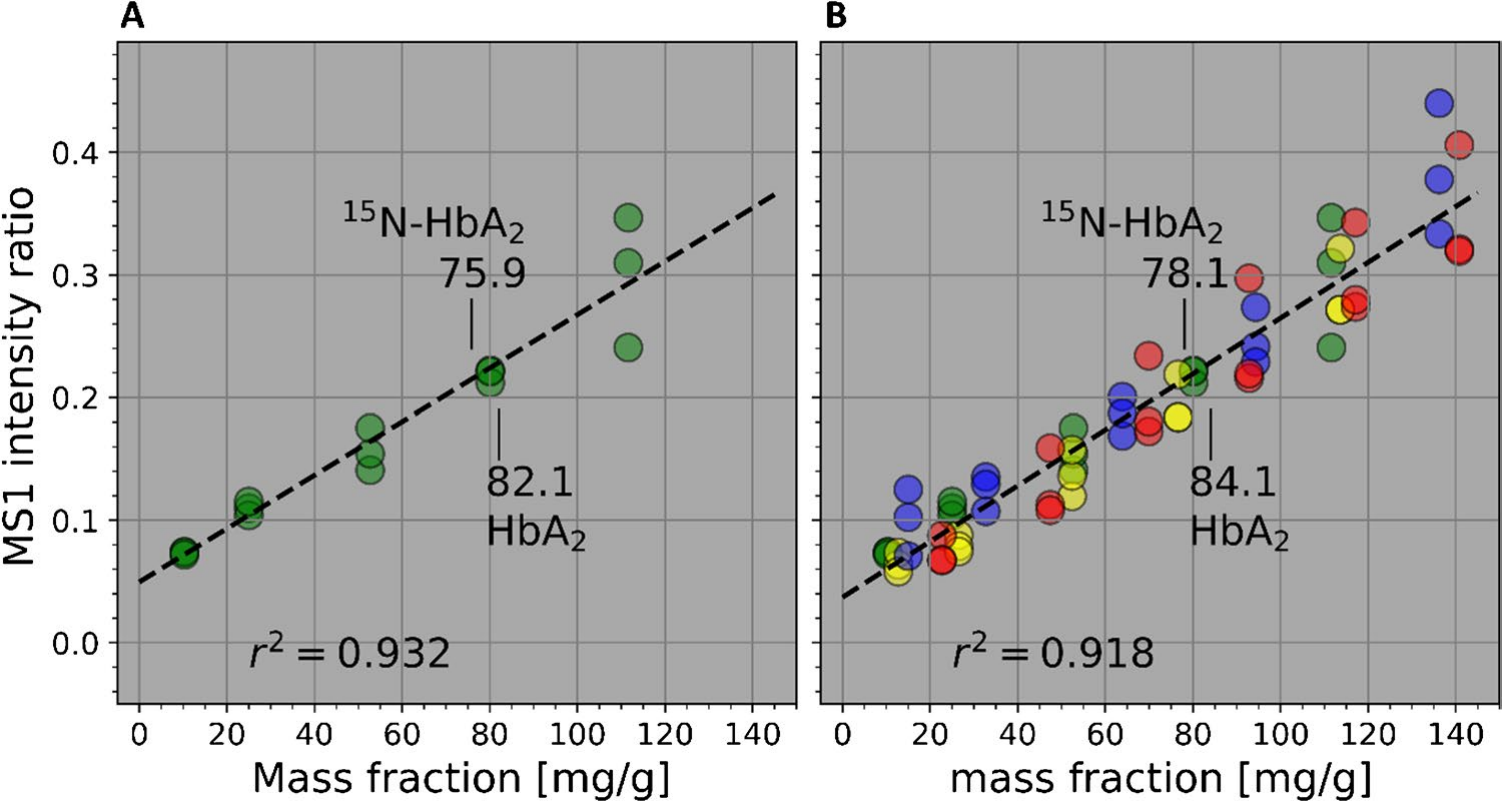
Calibrating MS1 intensity ratios (proteome ÷ HbA_2_) vs. mass fractions of HCPs impurity in HbA_2_. **A** Calibration using *E. coli* lysate. **B** Joint calibration using a set of different proteomes in addition to *E. coli*: *E. coli* lysate (green), yeast (yellow), human K562 cell line (blue), and a mix of nine neat proteins (red). Dashed lines: linear regression fit. Annotations: results for the HbA_2_ material and the U-^15^N-labeled HbA_2_ material using these calibrations

The mass fractions of *E. coli* in the HbA_2_ (raw) products (natural and labeled HbA_2_ materials), as well as in the ultra-purified HbA_2_ material, were quantified based on a standard shotgun proteomics approach, as illustrated in Fig. 1. MS1 intensity ratios (*E.coli* ÷ HbA_2_) were calibrated against known mass fractions of *E. coli* proteins relative to HbA_2_ (Fig. 1A and D). In this way, HbA_2_ technically also provides a pseudo internal standard (PIS), [5] improving the reproducibility of the measurements. For calibration, a linear model was established for the dependence of the instrumental response (MS1 intensity ratio) on the mass fraction of proteins. In turn, as is common in quantitative chemistry, this model function was resolved for the fraction as a dependent variable, thus allowing the fraction to be predicted from an intensity ratio as input (red arrows in Fig. 1D). The result of such a calibration with *n* = 3 repeats at each level, and subsequent value assignment to both HbA_2_ materials (natural and labeled) is shown in Fig. 2A.

### Representation of the studied materials by the E. coli lysate

**Fig. 3:**
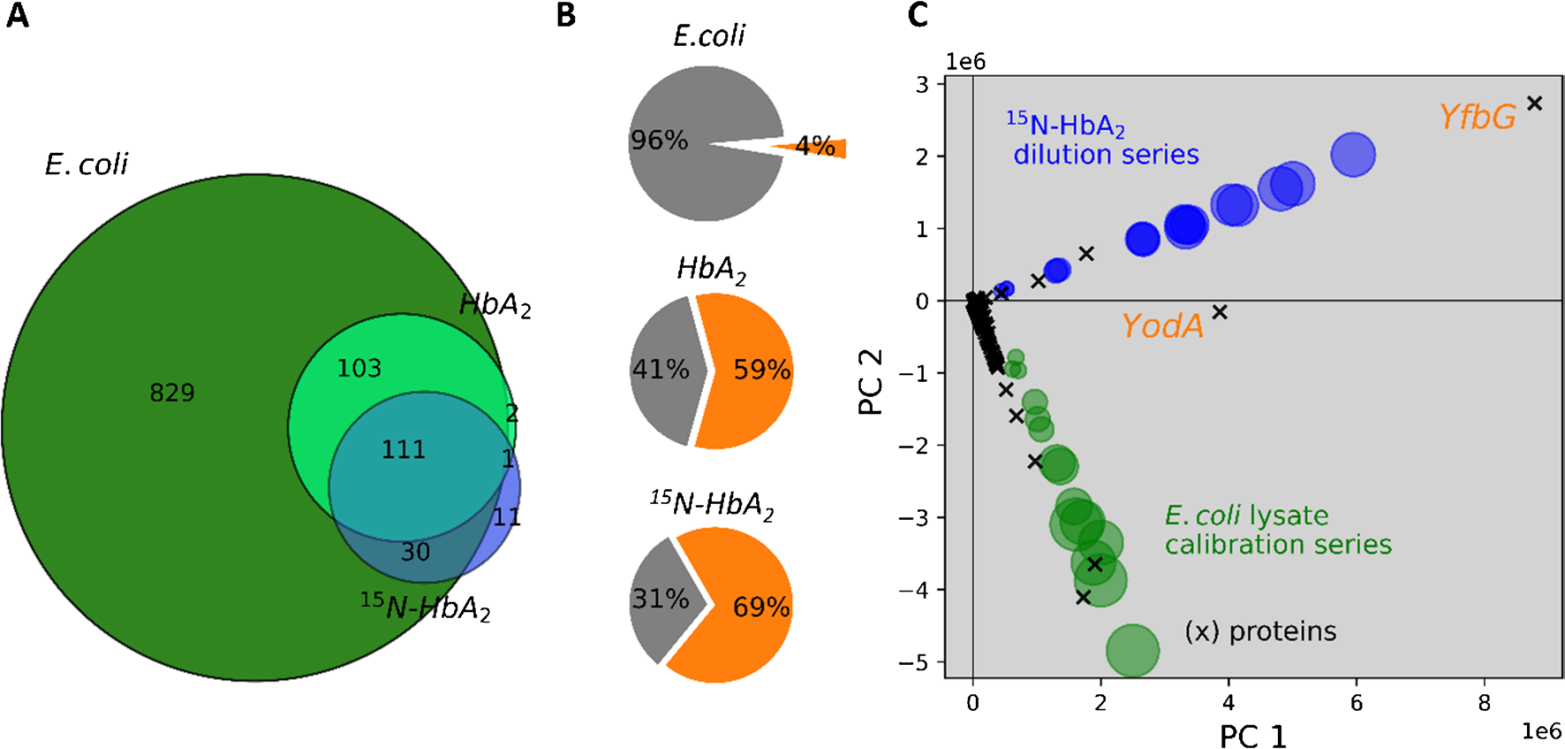
Comparison of the *E. coli* protein profile in the lysate with the HbA_2_ materials. **A** Identifications. **B** Relative amounts we found in these samples of frequently being co-purified [13] *E. coli* proteins (orange). **C** Interrelation of *E. coli* protein fractions in *E. coli* lysate (green circles) and in labeled HbA_2_ material (blue) with MS1 intensities of individual proteins (x), illustrated by a principal components plot. The series of protein fractions for the lysate is based on the same data source as the calibration data shown in Fig. 2A and B (green subset), whereas the HbA_2_-series was obtained by dilution of the labeled HbA_2_. Areas of the circles are in proportion with the fractions in the pertaining samples. The plot is a projection of the data on the plane of the first two principal components, PC 1 and PC 2, of the joint dataset (lysate plus labeled HbA_2_). Variance coverage: 61.6% (PC 1) and 36.1% (PC 2)

The impurity protein profiles in the studied materials differ significantly from those in the whole cell lysate. This is illustrated by different aspects in Fig. 3. First, as expected, the set of identified *E. coli* proteins in the materials is significantly reduced relative to the whole cell lysate (Fig. 3A). Many of these are known to typically be co-purified, if using IMAC for cleanup. Particularly, YfbG (P77398), YodA (P76344), GlmS (P17169), and ArgE (P23908) correspond to proteins previously reported in this context [13]. They make up a fraction of 60–70% (orange in Fig. 3B) in the studied materials, but represent only about 4% in the whole cell lysate. The difference in protein profiles is further substantiated by a principal component plot of results, as shown in Fig. 3C. Two series of samples of systematically changed *E. coli* mass fractions are shown. The first one is simply the same data as was acquired with the *E. coli* lysate calibration (green in Figs. 2A, 3A, and C). The second one was generated by dilution of the labeled HbA_2_ material (153 proteins, blue in Fig. 3A and C). Unlike with most applications of principal component analysis (PCA), no data scaling was applied for the results in Fig. 3C. Object scores (samples at different levels of mass fraction) and feature loadings (*E. coli* proteins quantified) are jointly shown in the space of the first two components (PC1 and PC2). In this kind of presentation, the proximity of a protein (small black crosses) to a series (blue or green circles) corresponds with the involvement of that protein in the MS1 signal ratio for that series. At the same time, the distance from the origin quantitatively reflects the degree of this involvement. Visibly, the majority of proteins are significantly involved in just one of both materials. This particularly holds for the top abundant ones, such as YfbG. As an exception, on the other hand, YodA is one of the few markedly involved in both materials, though not to exactly the same extent.

**Fig. 4:**
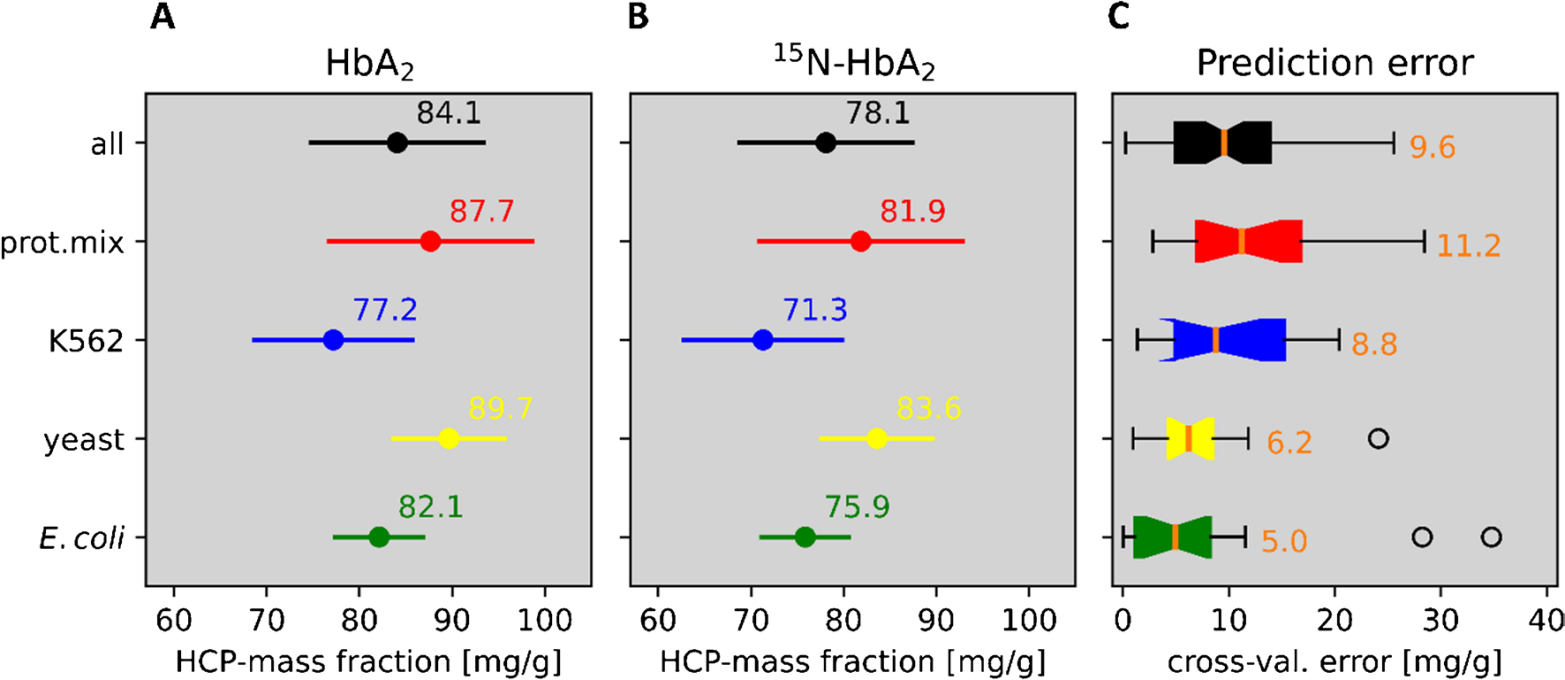
HCPs fractions determined by LFQ for both HbA_2_ materials, and degree of equivalence between different calibrator sources: *E. coli* lysate (green), yeast (yellow), human K562 cell line (blue), protein mix (red), and all of these series merged into one (black). **A** Natural isotopic HbA_2_ material and **B** isotope labeled version; **C** distribution of prediction error obtained by leave-one-out cross-validation of the calibration data; orange lines and numbers provide the medians. Circles: fliers. Error bars in (**A**) and (**B**) correspond to the medians in (**C**)

The demonstrated difference in protein profiles obviates prediction of impurity in an unknown material by linear regression models using as inputs the individual proteins from another material (as e.g., *E. coli*). As previously mentioned, LFQ works around the problem by integrating signals over all proteins for *E. coli* on one hand and in relation to HbA_2_ on the other, in order to map the mass fraction. This notion, indeed, has been the consensus in the literature for some time, [22–24] but still was put to the test for the present purpose. Moreover, additional proteomes, beyond *E. coli* lysate, were used for calibration, but were otherwise subjected to the same workflow as before. These proteomes were yeast, K562, and a mix of nine neat proteins. The eventual reduction to the simple protein mix was on purpose to provide an artificial proteome with a minimum number of components. Results of the respective calibration runs are plotted in Fig. 2B; HCPs mass fractions obtained for the two HbA_2_ materials by application of these calibrations are annotated in Figs. 4A and B. Apparently, there is good agreement on the whole between the individual plots. This supports the assumption that the individual linear calibration models (per proteome) are samples from a common statistical population. This in turn suggests that calibration based on the *E. coli* lysate (Fig. 2A) should essentially be valid for predicting HCPs fractions in the studied materials, too. Finally, pooling all individual calibrations into a common one is possible, as shown in the top trace (black) in Fig. 4, which may enhance the statistical robustness of value assignments.

### Overall uncertainty

The reasonably identifiable major sources of uncertainty are associated with (i) mass fractions assigned to the calibrators (Fig. 1A) and (ii) repeatability of sample preparation, calibration, and measurement (Fig. 1B and D). Treating the underlying random variates as independent of one another, and referring to *U*_*cal*_*, U*_*meas*_ as expanded relative uncertainties, [25] the resulting overall uncertainty on the impurity is *U *^*2*^_*imp*_ = *U *^*2*^_* cal*_ + *U *^*2*^_*meas*_*.*

The calibrator uncertainties (i), *U*_*cal*_*,* are likely to be dominated by value assignment of mass fractions to the calibrator stock solutions (HbA_2_, *E. coli* lysate, yeast, K562, and protein mix). ID-MS-based amino acid analysis (AAA) was employed for this, with 3.5% uncertainty or less at 95% confidence (see “Experimental section”, “Determination of protein mass fractions in the calibrators”).

The uncertainty contribution by sample preparation, sample measurement plus establishment of the calibration was estimated from the results of calibration measurements according to a common approximation; see, e.g., [26], chapter 5. For the present purpose, the set of *n* = 18 calibration results for the protein mix was used. The reference to the protein-mix calibration is motivated by the fact that the prediction error was highest with the protein mix compared to the other calibrator sources, consequently providing us with a conservative estimate. As the outcome does not only depend on the calibration results, but also on the number of sample measurements that were averaged to calculate the result, two different standard uncertainties were obtained: *u*_*meas*_ = 16.7% for the natural HbA_2_ -material (with just one measurement), while *u*_*meas*_ = 8.3% for the labeled one, as based on six measurements. With *k* = 2.12 (using the student’s *t* value for 16 degrees of freedom), the expanded uncertainties, *U*_*meas*_, are 35.4% for the natural and 17.6% for the labeled material, resp. Combining uncertainty components *U*_*cal*_* and U*_*meas*_*,* eventually yields *U*_*imp*_ = 35.6% and 18.0%, resp. Based on this, the impurity mass fractions are 84 ± 30 mg (impurity)/g(HbA_2_) in the natural and 78 ± 14 mg (impurity)/g(HbA_2_) in the labeled material. In terms of purity, this makes 923 mg(HbA_2_)/g(total protein) with a confidence interval of 898–949 mg/g and 928 mg(HbA_2_)/g(total protein) with an interval of 916–940 mg/g. This corresponds to about ± 2.8% and ± 1.3%, resp., in terms of relative uncertainty on the purity values.

### Result for the ultra-purified HbA_2_ material

To demonstrate the scalability of the method, a separate calibration was performed in the range of 0.1–1.5 mg/g fraction of protein mix. Based on duplicate measurements of calibrators at 0.15, 0.3, 0.43, 0.71, 1.08, and 1.42 mg/g, a linear fit was obtained at a coefficient of determination of *r*^2^ = 0.964, comparable to the previous broader-range calibrations (as, e.g., 0.932 with *E. coli*, cf. Figure 2A).

By a single sample measurement based on the established calibration, in the same way as above, *u*_*meas*_ = 11.6% and *U*_*meas*_ = 25.8% (*k* = 2.23) were obtained and combined with U_cal_ (3.5%) to the overall uncertainty *U*_*imp*_ = 26.1%. This results in 0.86 ± 0.22 mg(impurity)/g(HbA_2_), corresponding to 999.1 mg(HbA_2_)/g(total protein) for the HbA_2_ fraction, or purity, resp., with a confidence interval of 0.9989–0.9994 mg/g. This is equivalent to 0.02% uncertainty.

### Sample size and associated error

**Fig. 5:**
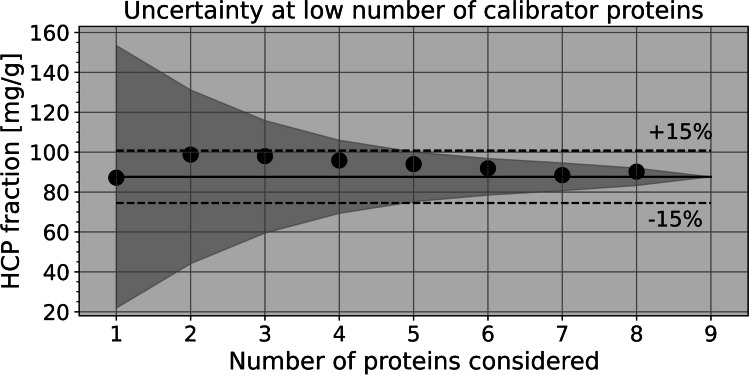
Estimating the sampling error caused by the finiteness of the number of proteins/peptides used for calibration or present in the sample. The data shown are results for the HbA_2_ material after stepwise reduction of the number of proteins included in calibration with the protein mix. Solid line: median obtained at *n* = 9 (87.7 mg/g), dashed: ± 15%. Scatterpoints: median results after recalculating the calibration function using random drafts of *n* = 1–8 out of the originally nine proteins. Dark grey area: corresponding standard deviations (shown here relative to the solid line, rather than to the medians)

The previous results suggest that calibration can in practice be performed with a small number of well-characterized proteins just as well as with complex biological materials. However, LFQ depends on the assumption that the peptides captured by their MS1 intensities are models from the same population, for sample and calibrator, as regards molar sensitivities. Consequently, on significantly reducing the number of peptide species involved, an associated sampling error will become apparent, thus increasing the overall measurement uncertainty. In Fig. 5, results of a simulation are shown that seeks to estimate the size of that potential error. Calibration based on the protein mix was used and the deviation of obtained HCPs mass fraction for the (unlabeled) HbA_2_ material calculated, assuming a reduction in the number of proteins used for calibration down to *n* = 8 − 1 proteins, randomly selected from the nine. To generate a distribution of possible outcomes, 100 random drafts of this number of proteins were acquired at each level, and the respective results for the HCPs mass fraction were calculated. The data shown in Fig. 5 cannot exactly map reality, of course, since, even if using all of the nine proteins, the sampling error will be less than with just one, but cannot completely disappear at *n* = 9. As such, Fig. 5 does not exactly reflect the ground truth, but it should be close. Accepting this, the example suggests that a number of five proteins may suffice on average to keep the sampling error at 15% or less.

### Top-N protein quantification strategy as an alternative

In the introduction, we claimed an exhaustive one-by-one quantification of individual HCPs to be non-practical if the aim is to find the total amount (or fraction) of HCPs in cell-expressed isolated proteins. Revision of the data shown in Fig. 3C indeed suggests the option of individually quantifying the top 5 *E. coli* proteins (YfbG and YodA for the most abundant ones) and taking the sum as an estimate.

However, integrating the MS1 signals over these five proteins results in only 69% of what was obtained if integrating over all proteins (as proposed in this study) for the natural material and 88% for the labeled one. In terms of impurity fraction, this would be a systematic error of 31% and 12%, resp., caused by non-captured proteins, resulting in overestimation of material purity by about 2.5% for the natural HbA2, and about 0.9% for the labeled one. Depending on the intended use, this added systematic uncertainty may still be acceptable. However, a further argument in favor of LFQ is that the one-by-one approach is likely to be more expensive, compared to a series of simple shotgun-experiments, as required in LFQ.

## Conclusions

LFQ is applicable to the quantification of host cell-derived impurity in bioengineered proteins. Calibrating the integrated MS1-intensity for all HCPs against the same quantity obtained for samples of known mass-fractions is a straightforward solution to the problem of quantitatively capturing a composite set of individual proteins ultimately to be expressed as a gross-measurand. The viability of this process is not hampered by the fact that the profile and identities of HCPs do not normally coincide with those of the calibrator material. This opens up the option of using proteomes for calibration other than those suggested by the expression system. This commutability of materials means that simple mixtures of well-characterized proteins are also viable candidates.

For the natural HbA_2_ material, we estimate 84 ± 30 mg(impurity)/g(HbA_2_), for the isotope-labeled HbA_2_ material 78 ± 14 mg/g and for the ultra-purified (natural) HbA_2_ material 0.86 ± 0.22 mg/g. This translates to 923 ± 2.8%, 928 ± 1.3%, and 999.1 ± 0.02% mg(HbA_2_)/g(total protein), respectively, fractions of HbA_2_ in the materials. The latter provide the correction factors to be applied to a quantitative result, if using these materials as a reference. For the first two (IMAC-purified) materials, an uncertainty result of ≈1.3–3.0% contributed to the overall budget for the analytical result, while ≈0*.*02% was obtained for the ultra-purified material. Considering the expense in terms of material loss, and assuming a target of 5% uncertainty as acceptable for the protein as a measurand in a biological sample, immediate use of the IMAC-purified material would have been optimal when compared to the efforts required for further purification.

Although discussed here in the context of value-assignment to materials to be used as primary calibrators with protein quantification, LFQ is increasingly also being used in areas, such as process optimization and quality control of pharmaceutical products [15, 27–31]. Typically in most of these applications, it is about quantification of individual proteins, rather than aiming at a mass fraction as a whole for HCPs. However, capturing a mass fraction of HCPs as a gross quantity, as discussed in this paper, or selectively for protein-subclasses of particular interest, could gain importance in these industries for reasons of particular toxicity of such classes, or other legal requirements.
